# IRAK-M Expression Limits Dendritic Cell Activation and Proinflammatory Cytokine Production in Response to *Helicobacter pylori*

**DOI:** 10.1371/journal.pone.0066914

**Published:** 2013-06-11

**Authors:** Jessica Shiu, Steven J. Czinn, Koichi S. Kobayashi, Yezhou Sun, Thomas G. Blanchard

**Affiliations:** 1 University of Maryland School of Medicine, Baltimore, Maryland,United States of America; 2 Texas A&M College of Medicine, College Station, Texas, United States of America; 3 Institute for Genomic Sciences, University of Maryland School of Medicine, Baltimore, Maryland, United States of America; Oklahoma Medical Research Foundation, United States of America

## Abstract

*Helicobacter pylori* (*H. pylori*) infects the gastric mucosa and persists for the life of the host. Bacterial persistence may be due to the induction of regulatory T cells (T_regs_) whichmay have protective effects against other diseases such as asthma. It has been shown that *H. pylori* modulates the T cell response through dendritic cell reprogramming but the molecular pathways involved are relatively unknown. The goal of this study was to identify critical elements of dendritic cell (DC) activation and evaluate potential influence on immune activation. Microarray analysis was used to demonstrate limited gene expression changes in *H. pylori* stimulated bone marrow derived DCs (BMDCs) compared to the BMDCs stimulated with E. coli. IRAK-M, a negative regulator of TLR signaling, was upregulated and we selectedit for investigation of its role in modulating the DC and T cell responses. IRAK-M^−/−^ and wild type BMDC were compared for their response to *H. pylori*. Cells lacking IRAK-M produced significantly greater amounts of proinflammatory MIP-2 and reduced amounts of immunomodulatory IL-10 than wild type BMDC. IRAK-M^−/−^ cells also demonstrated increased MHC II expression upon activation. However, IRAK-M^−/−^ BMDCs were comparable to wild type BMDCs in inducing T-helper 17 (T_H_17) and T_reg_ responses as demonstrated in vitro using BMDC CD4+ T cells co-culture assays,and in vivo though the adoptive transfer of CD4^+^ FoxP3-GFP T cells into *H. pylori* infected IRAK-M^−/−^ mice. These results suggest that *H. pylori* infection leads to the upregulation of anti-inflammatory molecules like IRAK-M and that IRAK-M has a direct impact on innate functions in DCs such as cytokine and costimulation molecule upregulation but may not affect T cell skewing.

## Introduction

*Helicobacter pylori (H. pylori)* colonizes the gastric mucosa of over half of the world's population [1]. Infection lasts for life and is associated with a variety of gastric diseases including peptic ulcer disease, gastric adenocarcinoma, and MALT lymphoma [1]–[7]. Greater than 80% of infected people do not develop disease but even asymptomatic individuals develop histologic gastritis [8], [9]. The lack of disease in most individuals was originally believed to be due in part to variations in bacterial virulence mechanisms between *H. pylori* strains. It is becoming increasingly evident however that limited disease is due in large part to host immunoregulatory mechanisms, a response that also favors bacterial persistence[10]–[17].

The development of histologic gastritis is T cell-dependent and is predominantly driven by a mix of T_H_1 and T_H_17 responses [18]–[23]. Despite the role of these T helper subsets in promoting inflammation, it has been shown that regulatory T cells (T_regs_) accumulate in the gastric mucosa during chronic *H. pylori* infection and contribute to persistent *H. pylori* colonization [10], [13]–[15], [17]. The loss of regulatory T cell function in murine models of *Helicobacter* infection results in significantly increased inflammation and reduced bacterial loads, demonstrating that these *H. pylori*-mediated immunomodulatory effects may be beneficial to the host and the bacteria[10], [15], [16]. The benefits to the host extend beyond the stomach as *H. pylori* infection has been inversely correlated with esophageal cancer in adults and wheezing in children. The protective effects of *H.pylori* infection maybe dependent on T_regs_ [24]–[27].

Down regulation of the host immune response is mediated by regulatory T cells but the bacterial, environmental, and cellular factors that promote the activation of regulatory T cells remain ill-defined for *H. pylori* infection. Dendritic cells (DCs) are potent antigen-presenting cells that are critical for the induction of downstream adaptive immune responses [28], [29] and they have been demonstrated to play an important role in *H. pylori* infection. DCs sense *H. pylori* primarily through Toll-like receptors (TLR) 2 and 4 in a MyD88 dependent manner [30], [31]. *H. pylori* infection however may skew the DC response to favor the generation of T_regs_ cells via IL-18 dependent mechanisms [12], [27]. This Treg response, influenced by DCs, also protects against asthma in mice [32].

A better understanding of how *H. pylori* affects DC function and how DCs regulate downstream immune events may provide additional insight into *H. pylori* pathogenesis and persistence but may also enhance our understanding of the host response to mucosal bacteria in general. One of the mechanisms employed by the host to limit microbial induced activation of APCs is the expression of interleukin-1 receptor–associated kinase M (IRAK-M), a negative regulator or TLR [33]. IRAK-M expression has been demonstrated to limit immune activation to specific pathogens, and to play a role in maintaining immune homeostasis in the gut through its inducement by commensal bacteria [33]–[36]. We observed upregulation of IRAK-M in a transcriptome analysis of *H. pylori* stimulated DCs, one of only ten genes to be induced. The purpose of the present study therefore was to characterize the role of IRAK-M in *H. pylori*-activated DCs and to determine whether IRAK-M influences activation of the T cell response.We now report that IRAK-M expression in DCs is dependent upon TLR activation, and its expression is associated with limiting the innate proinflammatory activity of the DC, as well as maturation as measured by MHC II expression.

## Methods

### Ethics Statement

This study was carried out in strict accordance with the recommendations in the Guide for the Care and Use of Laboratory Animals of the National Institutes of Health. The protocols were approved by the Institutional Animal Care and Use Committee of the University of Maryland in Baltimore (#0809004). All efforts were made to minimize pain and suffering.

### Mice

Six- to thirteen-week-old C57BL/6, TLR2^−/−^, and TLR4^−/−^ mice were obtained fromJackson Laboratory (Bar Harbor, ME). IRAK-M deficient mice were derived on a C57BL/6 background as described previously [33] and were bred homozygously at the University of Maryland School of Medicine (Baltimore, MD, USA). C57BL/6 FoxP3-GFP mice and C57BL/6 OT-II Foxp3-GFP mice were a generous gift from David Scott (Bethesda, MD, USA) [37]. All animals were housed under pathogen-free conditions in microisolater cages at the University of Maryland Baltimore animal facilities. Mice were euthanized for tissue collection by CO_2_ asphyxiation followed by thoracotomy.

### Bacterial Strains and Infection

*E. coli* K12 was purchased from ATCC (#29425) (Manassas, VA) and grown on LB plates supplemented with amphotericin B (2.5 µg/ml). The mouse-adapted *H. pylori* Sydney Strain 1 (SS1) [38]and strain 26695 (ATCC #700392) were grown on Columbia agar (Difco, Detroit, MI) supplemented with7% horse blood and antibiotics at 37°C. For inoculation of mice, bacteria were transferred to 10 ml Brucella broth (Difco) supplemented with 10% fetal bovine serum (Invitrogen, Carlsbad, CA) and amphotericin B (2.5 µg/ml). Liquid cultures were established in T25 flasks and maintained at 37°C with 10% CO_2_. Infections with *H. pylori* SS1 were performed by delivering 1×10^7^ CFU in 0.5 ml Brucella broth by oral gavage using a 20 G feeding needle attached to a 1cc syringe. Antigen lysates were prepared as previously described [39].

### Generation of BMDCs and in Vitrostimulation Assays

Femurs and tibias were removed from 6–14 week old C57BL/6 WT, TLR2^−/−^, TLR4^−/−^, and IRAK-M^−/−^ mice at necropsy. Bone marrow was flushed out with a syringe filled with RPMI 1640 and cells were cultured in RPMI medium supplemented with either 100 ηg/mL Flt3L (R&D Systems, Minneapolis, MN) or 7 ηg/ml GM-CSF, and 10% heat inactivated FBS. Bone marrow derived DC (BMDC) were recovered after 8–9 days and plated in 48 well plates at 1×10^6^ cells/well. Stimulation of BMDCs was performed with 10 µg/mL of either *H. pylori* SS1 lysate, *H. pylori* 26695lysate or *E. coli* K12 lysate. For stimulation with live bacteria, bacterial density was determined by optical density at 450 ηm and used at a multiplicity of infection (MOI) of 10. Supernatants were collected at 4, 8, and 24 hours after addition of lysate for determination of TNFα, IL-10 and MIP-2 levels by quantitative enzyme linked immunosorbent assay (ELISA) using the relevant Duoset kits according to the manufacturer's instructions (R&D Systems).

### T Cell Co-culture Experiments

Coculture experiments were performed by plating 1×10^5^ BMDCs per well in 90 well U-bottom plates and stimulating with 10 µg/mL OVA peptide. CD4^+^ T cells were isolated from spleens from 6–14 week old C57BL/6 OT-II Foxp3-GFP mice using the CD4^+^ MagCellect Isolation Kit (R&D Systems) according to the manufacturer's instructions. T cells were added 5×10^5^ cells per well to the BMDC in 96 well plates in the presence of either T_reg_ promoting conditions (20 ng/mL TGF-β (R&D Systems) & 25 U of mIL-2 (E-bioscience, San Diego, CA), or T_H_17 promoting conditions (2 ng/mL TGF-β (R&D Systems) & 20 ng/mL mIL-6 (Gemini Bio-products, Sacramento, CA). Alternatively, 1×10^5^ BMDCs were plated in 90 well U-bottom plates and stimulated with media alone or 10 µg/mL *H. pylori* SS1 antigen lysate. CD4^+^ T cells were isolated from spleens from 6–14 week old C57BL/6 mice infected with *H. pylori* SS1 and 5×10^5^ T cells were added to the wells in the absence of any additional stimulation.

### Flow Cytometry Analysis

T-cells were stained with anti-CD4-APC and anti-IL17A-PE (eBioscience). BMDCs were stained with anti-MHCII-Pacific Blue, anti-PD-L1 PE, anti-CD40 PE-Cy5, anti-CD86 PE-Cy5 (eBioscience). All cells were analysed using a LSRII flow cytometer (BD Biosciences, San Hose, CA). Data were analyzed by FlowJo7 software (Tree Star, Ashland, OR).

### Adoptive Transfer Experiments

CD4+ T cells were isolated from the spleens of FoxP3-GFP mice using the MagCellect Mouse CD4+ T cell isolation kit (R&D Systems) and sorted for GFP negative cells using a BD FACSAria flow cytometer. A total of 2×10^6^ CD4^+^, GFP^−^ cells were transferred into WT and IRAK-M^−/−^ recipients by tail vein injection. Animals were infected with SS1 on day 3 and animals were harvested at 8 weeks for analysis. RNA was isolated from gastric tissue using the RNeasy kit (Qiagen, Germantown, MD) and converted to cDNA for qPCR analysis of GFP expression.

### RNA Isolation, Microarray Processing and Microarray Statistical Analysis

BMDCs were recovered following stimulation at the indicated timepoints and RNA was isolated using the RNeasy kit (Qiagen). cRNA was prepared from RNA using the Illumina TotalPrep RNA amplification kit. cRNA samples were hybridized onto Illumina MouseRef8_v2.0 bead array chips (San Diego, CA) containing 25,967 probes (n = 3 for antigen lysate treated BMDCs, n = 2 for media treated BMDCs). Analysis was performed using the limma R package to compare gene expression profiles among different stimulated groups compared to media-treated BMDCs. Only probes with a false discovery rate (FDR) of <0.1 were included. Raw and normalized data sets were deposited into the NCBI Gene Expression Omnibus database under accession number GSE 44954.

### Quantitative real-time PCR

Quantitative PCR analysis was performed by first converting 1 µg RNA to cDNA using the Quantitect reverse transcription kit (Qiagen). cDNA was diluted 10-fold and PCR amplication was performed with an Eppendorf Realplex Instrument (Eppendorf AG, Hamburg, Germany) on 96 well plates with SYBR Green supermix (Fermentas, Glen Burnie, MD) in either duplicate or tripiclate. Each reaction mixture contained: 12.5 µL SYBR green mix, 11.1 µL dH_2_O, 0.2 µL each forward and reverse primers at 0.8 µM, and 1 µL cDNA. Primer sequences were as follows: IRAKM: TGAGCAACGGGACGCTTT (forward), GATTCGAACGTGCCAGGAA (reverse) [40]; GAPDH: CCAGGTTGTCTCCTGCGACTT (forward), CCTGTTGCTGTAGCCGTATTCA (reverse); GFP: CCTGAAGTTCATCTGCACCACC (forward), CTGCTGGTAGTGGT- CGGCGAGC (reverse). Relative gene expression changes were calculated using the ΔΔC_T_ method, and expression normalization was done using the housekeeping gene glyceraldehyde 3-phosphate dehydrogenase (GAPDH).

### Statistical Analysis

Statistical analysis was performed using GraphPad Prism for Macintosh 5.0 c (GraphPad Software, San Diego, CA). The Mann-Whitney test was used for analysis of statistical significance and a *P* value <0.05 was considered significant.

## Results

### *H. pylori* Stimulation of BMDCs Results in Gene Expression Changes in a Limited Set of Genes after 24 hours

**Figure 1:**
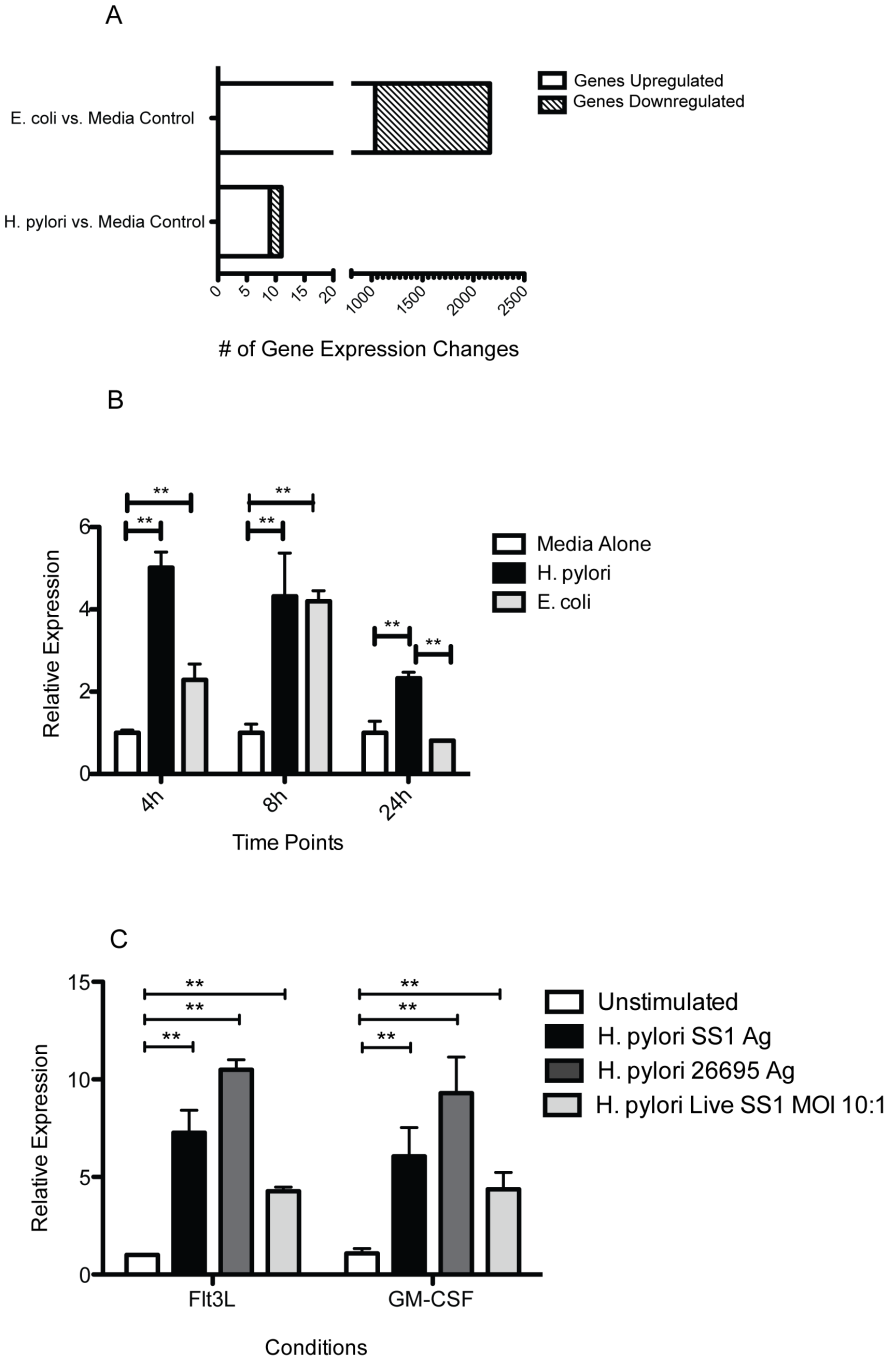
Global gene expression level changes in BMDCs stimulated with media alone, *H.* *pylori* or *E. coli* after 24 h. cRNA was hybridized onto Illumina Mouse Ref8_v2.0 chips with probes for >24,000 genes (n = 3 for antigen lysate treated BMDCs, n = 2 for media treated BMDCs). (A) The number of genes that were upregulated or downregulated in both treated groups compared to media alone. Data reflects probes with an FDR <0.1. (B) RT-PCR analysis confirmed that IRAK-M was significantly upregulated in BMDCS stimulated with either *H. pylori* or *E. coli* compared to media alone at 4 h, 8 h and 24 h. (C) At 24 h, both Flt3L and GM-CSF derived BMDCs upregulated IRAK-M expression after stimulation with either live SS1 bacteria (MOI 10), or SS1 and 26695 antigen lysate. **, *P*<0.01.

To identify the molecular pathways activated by *H. pylori* stimulation in DCs, we performed microarray analysis on BMDCs stimulated for 24 hours with either *E. coli* or *H. pylori* bacterial lysate. As expected, *E. coli-*stimulated BMDCs (EC-BMDCs) exhibited gene expression changes in many genes (Figure 1A). More than half of 2162 impacted genes were downregulated. Surprisingly, *H. pylori-*stimulated BMDCs (HP-BMDCs) showed limited gene expression changes with only 10 genes significantly affected in their expression (Table 1). Six of the 10 genes are implicated in anti-inflammatory pathways. Since *H. pylori* has been demonstrated to activate DCs primarily through TLR2 and TLR4 [30], [31], we were particularly interested in the increased expression of IRAK-M, a negative regulator of TLR signaling[33]. Using a qPCR approach, we confirmed that IRAK-M was indeed upregulated in BMDCs following activation with *H. pylori* lysate, and that in contrast to EC-BMDCs, IRAK-M expression in HP-BMDCs remained significantly high at 24 hour (*P*<0.01; Figure 1B). IRAK-M was demonstrated to be significantly upregulated in *H. pylori*-stimulated BMDC generated with either Flt3L or GM-CSF (*P*<0.01; Figure 1C) and this response was consistent with using lysates from either the Hp SS1 or the common laboratory strain 26695. Live Hp SS1 was also demonstrated to induce significant levels of IRAK-M expression although less effective than lysate antigen (*P*<0.01).

**Table 1:** *H. pylori* associated gene expression changes.

Gene Name	Fold Change(*H. pylori* vs. Media alone)
**Antimicrobial peptides**	
Elastase 2, neutrophil (Ela2)	−4.76
Cathelicidin antimicrobial peptide (CAMP)	−3.27
Lipocalin 2 (Lcn2)	2.09
**Anti-inflammatory molecules**	
Zinc finger CCCH type containing 12A (Zc3h12a)	1.46
Acyloxyacyl hydrolase (Aoah)	1.53
Interleukin-1 receptor-associated kinase 3 (Irak3/IRAK-M)	2.21
Nuclear factor of kappa light polypeptide gene enhancer in B-cells inhibitor, zeta (Nfkbiz/IκB-ζ)	2.71
Tribbles homolog 3 (Drosophila) (Trib3)	3.98
Vanin 3 (Vnn3)	4.06
**Trafficking Molecules**	
Vesicle transport through interaction with t-SNAREs homolog 1A (yeast) (Vti1a)	1.70

### Lack of IRAK-M in BMDCs Results in a More Pro-inflammatory Phenotype in HP-BMDCs

**Figure 2:**
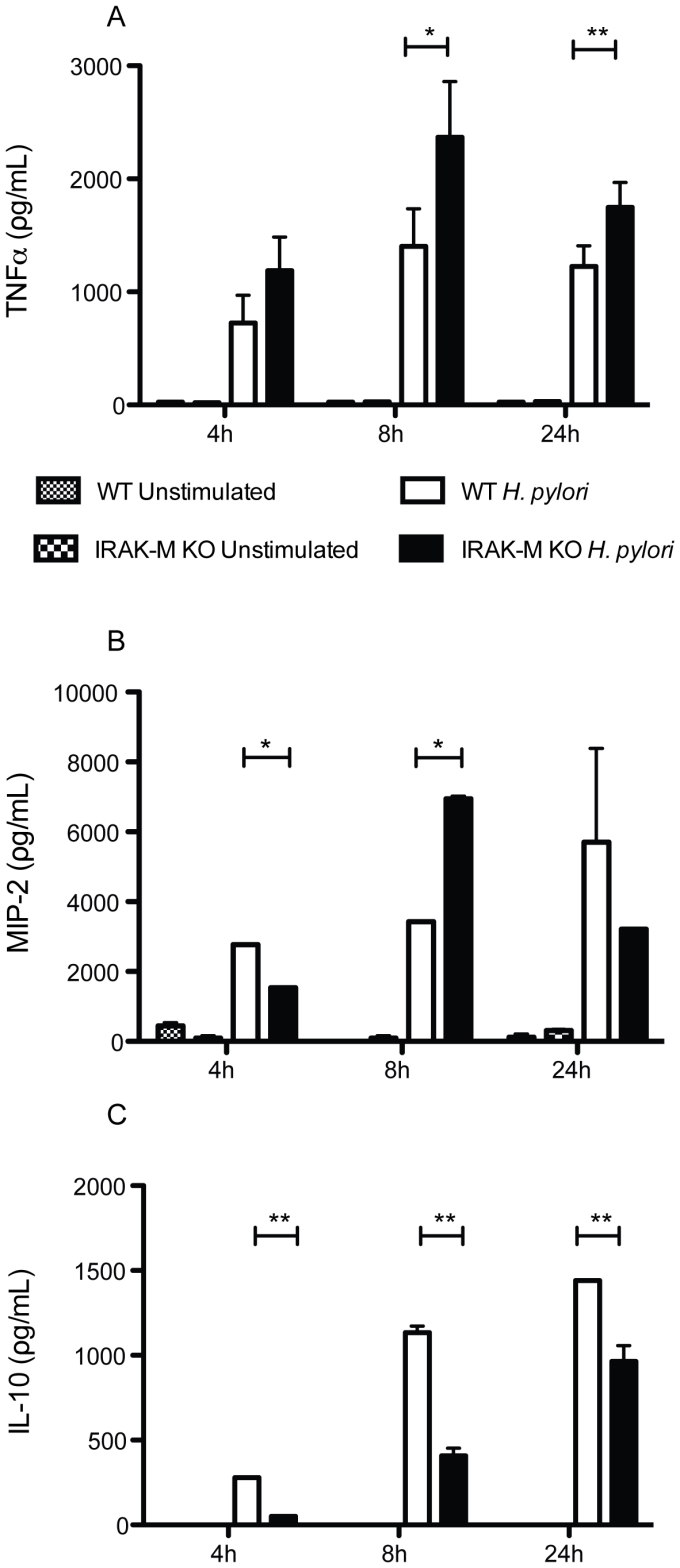
IRAK-M^−/−^ BMDCs exhibited a more proinflammatory phenotype after *H.* *pylori* stimulation compared to WT BMDCs. Supernatant from *H. pylori* antigen-stimulated WT and IRAK-M^−/−^ BMDCs were collected at 4 h, 8 h and 24 h and used to determine (A) TNFα, (B) MIP-2, and (C) IL-10 levels by ELISA. Data reflects three independent experiments. Error bars indicate standard deviations. *, *P*<0.05; **, *P*<0.01.

IRAK-M has been shown to play a role in DC activation in a tumor vaccine and in a LPS endotoxin tolerance model of *H. pylori* activation. [41], [42] Therefore, we wanted to determine if IRAK-M expression affects cytokine production in HP-BMDCs by comparing WT and IRAK-M^−/−^ BMDCs. IRAK-M^−/−^ cells have previously been shown to have a more proinflammatory phenotype [33], [41]. BMDCs deficient in IRAK-M responded to *H. pylori* antigens by producing TNFα and MIP-2 as early as four hours post activation and peaking at eight hours when levels of both cytokines were significantly higher than for WT BMDCs (*P*<0.05; Figure 2A and 2B; Supplemental Figure 1).TNFα levels remained higher in IRAK-M^−/−^ cells at 24 hours post stimulation (*P*<0.01). IL-12p70 was also measured but were undetectable (data not shown).This is consistent with our previous observations using H. felis activation of BMDC [43].Conversely, HP-BMDCs secreted significantly less IL-10 compared to WT HP-BMDCs at all time points although levels increased steadily over the 24-hour period (Figure 2C).

**Figure 3:**
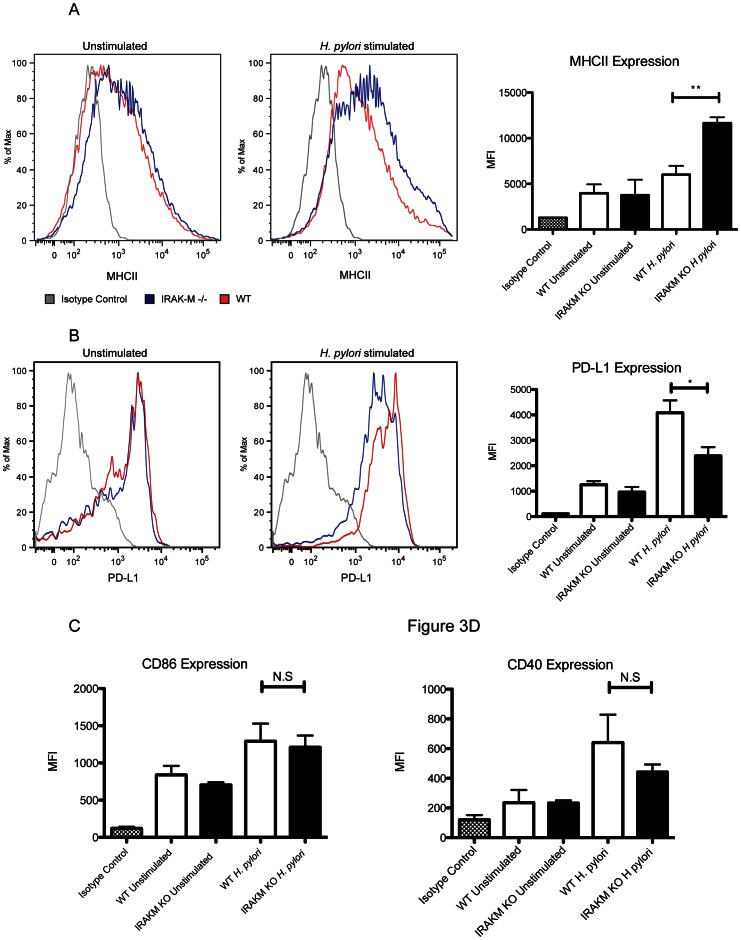
BMDCs stimulated for 24 h with *H.* *pylori* antigen were collected for flow cytometry analysis to determine (A) MHC-II expression and (B) PD-L1 expression compared to cells in media alone and isotype controls. Graphical representation of mean ± SD from data collected from three individual experiments performed in duplicate is shown on the right. Graphical representation of surface expression (C) CD86 and (D) CD40 determined by flow cytometry analysis *, *P*<0.05; **, *P*<0.01.

Cell surface analysis of activated cells showed that IRAK-M^−/−^ HP-BMDCs expressed higher levels of MHC II (*P*<0.01;Figure 3A), suggesting that IRAK-M normally limits DC activation as measured by MHC II expression in response to *H. pylori* stimulation. Conversely, expression of the down regulatory co-receptor PD-L1 was significantly reduced in activated IRAK-M−/− BMDC compared to WT cells (*P*<0.05; Figure 3B), indicating that IRAK-M normally limits the potential of DC to activate Th cells upon activation with *H. pylori*. Co-receptors CD86 and CD40 however remained comparable between activated IRAK-M−/− and WT BMDC (Figures 3C and 3D). Together, these data suggest that in response to *H. pylori* stimulation, IRAK-M expression contributes to a lack of DC maturation and promotes a regulatory phenotype exemplified by IL-10 production.

### IRAK-M upregulation in HP-BMDCs is dependent on both TLR2 and TLR4

**Figure 4:**
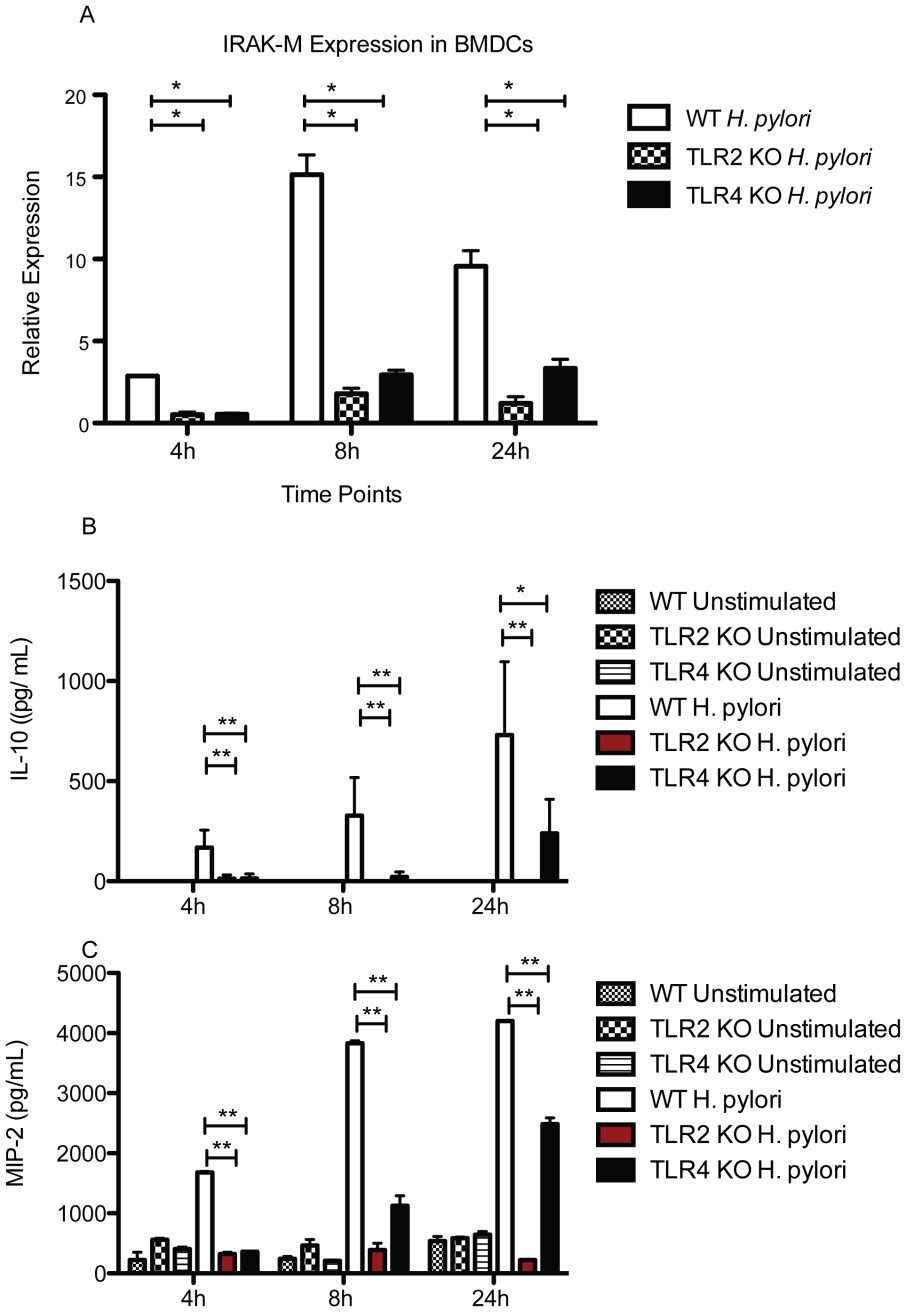
(A)IRAK-M induction in BMDCs after *H. pylori* stimulation is dependent on TLR expression. RNA from WT, TLR2^−/−^, TLR4^−/−^ BMDCs was collected at 4 h, 8 h and 24 h and converted to cDNA. qPCR was used to determine relative IRAK-M expression levels. The diagram represents mean ± SD from data collected from two individual experiments performed in duplicate. (B) Supernatants collected from *H. pylori* antigen-stimulated WT, TLR2^−/−^ and TLR4^−/^ BMDCs were collected at 4 h, 8 h and 24 h and used to determine (B) IL-10 and (C) MIP-2 levels by ELISA. *, *P*<0.05; **, *P*<0.01.

TLR2 and TLR4 have been shown to play an important role in *H. pylori* sensing by DCs [31]. We therefore sought to determine if either TLR2 or TLR4 might be important in IRAK-M upregulation by comparing HP-BMDCs from WT, TLR2^−/−^ and TLR4^−/−^ mice. Whereas WT HP-BMDC displayed a 15-fold increase in IRAK-M expression by eight hours that remained high at 24 hours, Figure 4A illustrates that IRAK-M upregulation in HP-BMDCs is dependent on both TLR2 and TLR4 expression, and that abrogation of either TLR results in a reduction in IRAK-M expression(*P*<0.05).Additional evidence for the importance of both TLR2 and TLR4 in *H. pylori* lysate induced activation of DC is demonstrated in Figure 4B and 4C where expression of IL-10 and MIP-2 respectively are shown to be significantly reduced compared to BMDC from WT mice (*P*<0.01). Although expression of both cytokines increased by 24 hours for TLR4 KO cells, these cytokines were largely absent in the cells from TLR2 deficient mice.

### IRAK-M expression in DCs does not affect T_H_17 differentiation in T cells

**Figure 5:**
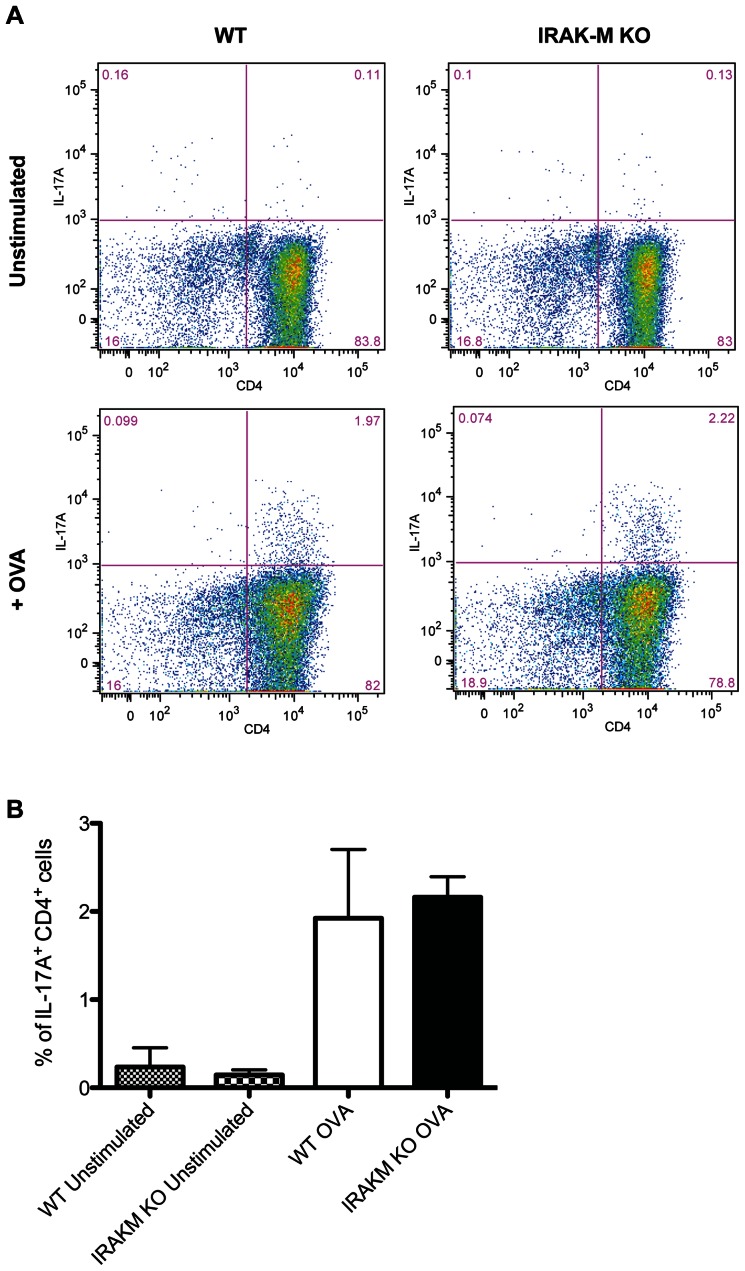
IRAK-M^−/−^ BMDCs do not increase TH_17_ induction in vitro. (A) BMDCs isolated from WT and IRAK-M^−/−^ mice were plated and pulsed with OVA for 2 hours before CD4+ T cells isolated from OT-II Foxp3-GFP animals were added to the wells in the presence of IL-6 and TGFβ for 72 hours. Cells were restimulated with PMA and ionomycin in the presence of monesin, and production of IL-17A in CD4^+^ T cells was measured by flow cytometry. (B) Data are representative of three independent experiments. Bar graph represents mean ± SD from data collected from three individual experiments performed in duplicate.

Since T_H_17 cells have been shown to contribute to the gastritis seen in *H. pylori* infection as well as to protection against *H. pylori* in experimental murine vaccine models [21], [44]–[46], we sought to determine whether the proinflammatory phenotype of IRAK-M^−/−^ BMDCs might increase T_H_17 activation using a DC-T cell coculture system. Studies using *H. pylori* stimulated BMDC cells to stimulate splenic CD4^+^ cells from mice infected with H. pylori showed no increase in either IFNγ or IL-17 producing cells from either WT or IRAK-M−/− mice (Supplemental Figure 2). This is consistent with the suppression that occurs in the *H. pylori*-specific T cell response in infected hosts. T cells from transgenic mice with a TCR specific for the OVA antigen were used to increase the frequency of responsive cells. IRAK-M^−/−^ BMDCs were similar to WT BMDCs in their ability to generate IL-17A^+^CD4^+^ T cells (Figure 5A and 5B). There was no difference in the number of IL-17A^+^ T cells following OVA exposure when *H. pylori* activated DC from WT and IRAK-M^−/−^ were used as APC cells.

### IRAK-M^−/−^ BMDCs are Comparable to WT BMDCs in Generating T_regs_

**Figure 6:**
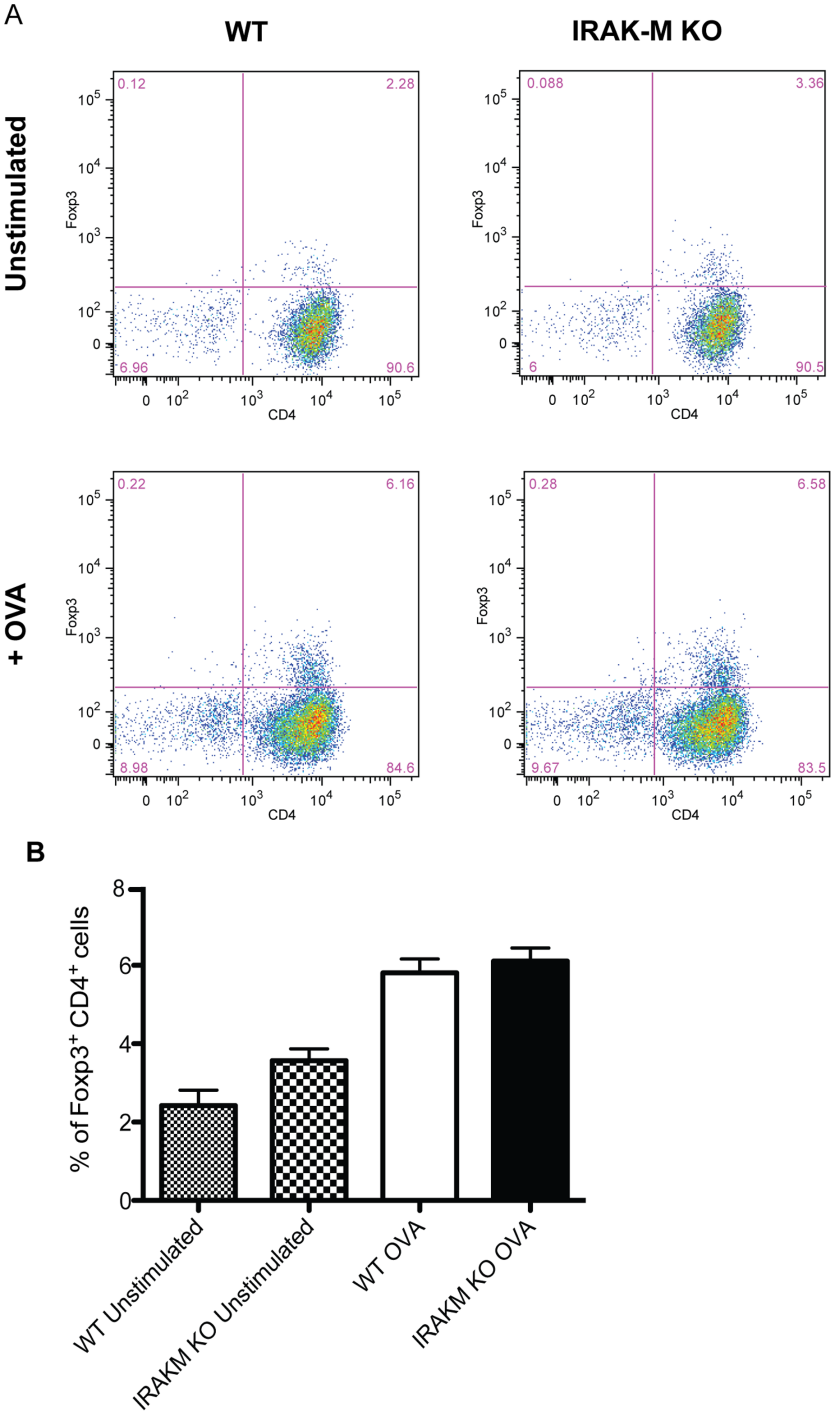
IRAK-M^−/−^ BMDCs do not affect T_reg_ induction in vitro. (A) BMDCs isolated from WT and IRAK-M^−/−^ mice were plated and pulsed with OVA for 2 hours before CD4+ T cells isolated from OT-II Foxp3-GFP animals were added to the wells in the presence of IL-2 and TGFβ for 72 hours. Cells were restimulated with PMA and ionomycin in the presence of monesin, and Foxp3-GFP expression in CD4^+^ T cells was measured by flow cytometry.Data are representative of three independent experiments. (B) Bar graph represents mean ± SD from data collected from three individual experiments performed in duplicate.

**Figure 7:**
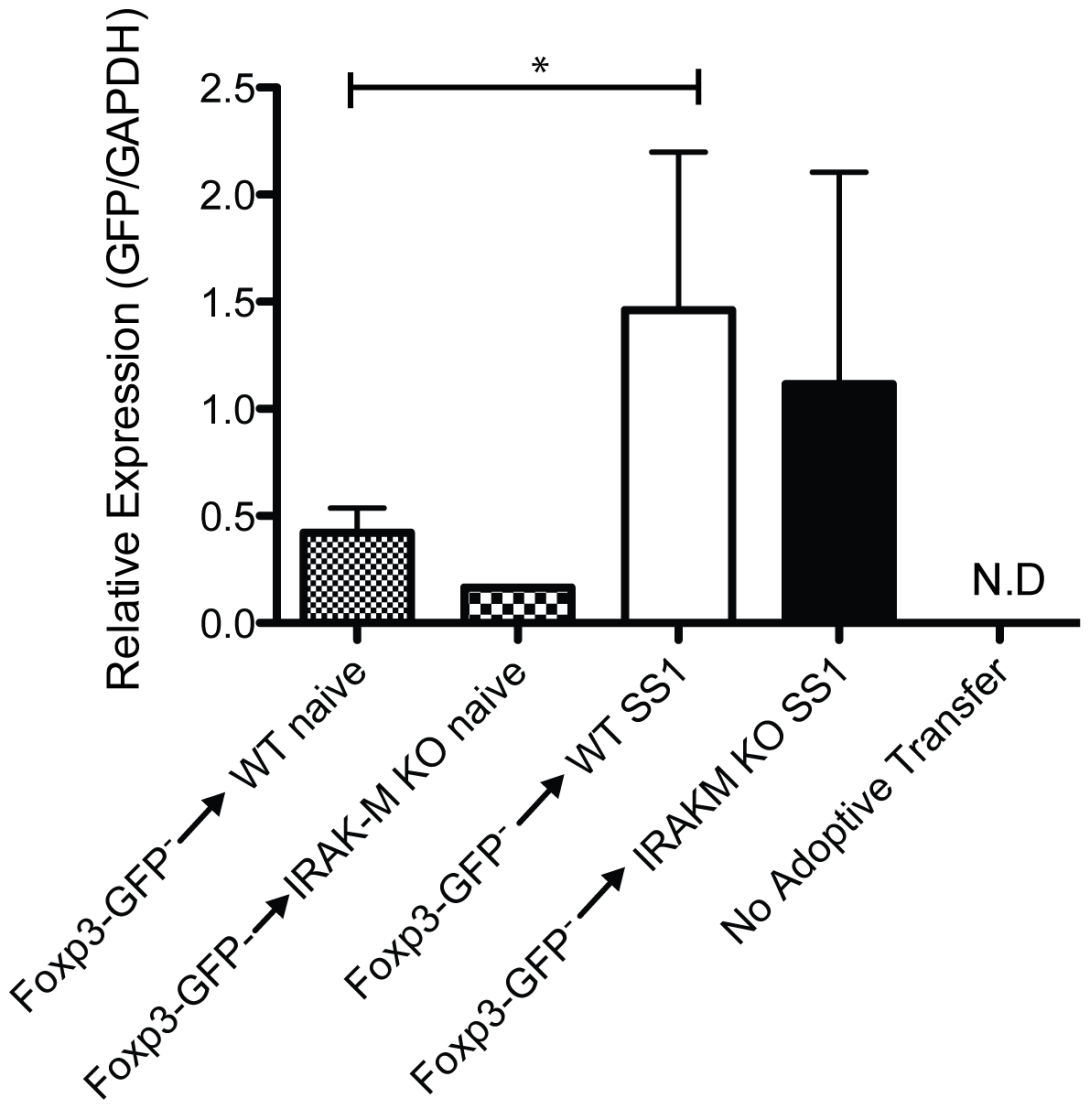
IRAK-M deficiency does not affect iT_reg_ generation in vivo. GFP^−^CD4^+^ T cells were isolated from Foxp3-GFP mice and sorted forlack of GFPexpression. 2×10^5^ GFP^−^ cells were transferred to the indicated recipient mice i.v. Mice were left untreated or infected with *H. pylori* SS1 for 8 weeks. RNA was isolated from gastric tissue to determine relative GFP expression in the stomach. Each group contained 3-6 mice. Bar graph represents mean ± SD. N.D  =  not detectable. *, *P*<0.05.

Since the balance of T_H_17/T_regs_ cells contributes to the extent of the inflammatory response in *H. pylori* infection [12], we also sought to determine if T_reg_ generation is affected by the lack of IRAK-M in BMDCs using the DC-T cell co-culture system described above. The OVA TCR transgenic mice are also transgenic of FoxP3-GFP expression, providing a convenient marker for FoxP3. HP-BMDC were co-cultured with these T cells and stimulated with OVA and the activated T cells were assessed by flow cytometery for GFP (Figure 6A and6B). WT and IRAK-M^−/−^ BMDCs did not differ in their ability to generate T_regs_. To determine whether IRAK-M expression influences T_reg_induction in response to *H. pylori* in vivo, we sorted CD4+ GFP^−^ T cells from Foxp3-GFP C57BL/6 animals to eliminate natural Treg cells and any preexisting iTreg cells. These GFP negative cells were used for adoptive transfer into WT and IRAK-M^−/−^ recipients. Recipient mice were subsequently infected with *H. pylori* and the amount of new FoxP3-GFP expression was determined four weeks later by isolating gastric tissue and using qPCR analysis. *H. pylori* infection in WT animals resulted in the induction of Foxp3 expression in the gastric mucosa (Figure 7, *P*<0.05). Gastric tissue from IRAK-M^−/−^ animals also had increased Foxp3 expression after *H. pylori* infection but levels were comparable to those observed in the gastric tissue of WT animals. Together, this data suggests that the proinflammatory phenotype of IRAK-M^−/−^ BMDCs does not affect T_reg_ generation.

## Discussion

Recent studies have demonstrated that DCs play an important immunoregulatory role in *H. pylori* infection and may even impact susceptibility or severity of other diseases such as asthma development [11], [12], [27], [32], [47]. An understanding of the molecular pathways that are activated in DCs by *H. pylori*, therefore, could provide significant insight into how immunoregulatory and inflammatory pathways are controlled during the course of infection and how these mechanisms may act more broadly. In the present study, we used a microarray approach to identify molecules in DCs whose expression is changed most significantly by *H. pylori*. We identified IRAK-M as a potential important regulatory protein for further characterization.

By comparing HP-BMDCs to EC-BMDCs in our microarray study, *H. pylori* appeared to be weakly immunogenic as only 10 gene expression changes were apparent after 24 hours. Although this contrasted significantly with the 2162 gene expression changes seen in the EC-BMDCs, our data are consistent with previous microarray analyses on *H. pylori*-activated cells. One study conducted a BMDC microarray following *H. pylori* exposure observed 126 gene expression changes after six hours [30]. A more recent study using *H. pylori* LPS stimulation of HEK293 cells reported only three significant gene expression changes after 24 hours [48]. The low number of gene expression changes may be a reflection of *H. pylori* pathogen associated molecular patterns (PAMPs)having reduced TLR stimulating activity and that after 24 hours, the induced expression of early genes may no longer be evident. The LPS is well documented to lack endotoxin activity, and structural studies confirm that the length and number of its lipid chains do not favor binding to TLR4 [49]–[51]. The flagellin protein has also been shown to lack the consensus sequence typically associated with binding to TLR5 [52].

Among the TLRs, TLR2 has been demonstrated to play a dominant role on the activation of APC by *H. pylori* [31]. We therefore investigated IRAK-M, a negative regulator of TLR signaling, in greater detail [33]. IRAK-M lacks kinase activity and its expression pattern was initially thought to be restricted to the monocyte/macrophage lineage [53]. It is now known that IRAK-M may also be expressed in dendritic cells and epithelial cells and is a key factor in endotoxin tolerance [33], [41], [54], [55]. IRAK-M^−/−^ BMDCs secrete increased levels of T_H_1 cytokines such as IFNγ, and skew the T cell response in vivotowards a more proinflammatory phenotype to prolong survival in a tumor vaccine model [41]. In a separate study, IRAK-M^−/−^ BMDCs that were tolerized to LPS exhibited higher levels of MHCII and lower levels of IL-10 [42].

In our study, IRAK-M^−/−^ BMDCs stimulated with *H. pylori* also displayed a more pro-inflammatory phenotype compared to WT BMDCs. IRAK-M^−/−^ BMDCs displayed increased MHC II expression and higher MIP-2, TNFα production, and also produced less immunoregulatory IL-10 and PD-L1. IRAK-M expression was shown to be dependent on TLR2 and TLR4 activation. Both TLR2 and TLR4 have been shown to be important in *H. pylori* infection [31]. Despite the impact of the IRAK-M deficiency on MHC II and cytokine balance in HP-BMDCs, IRAK-M^−/−^ DCs failed to change the balance of T cell subsets induced in vitro. DCs have been shown to affect the balance of T_H_17 and T_reg_ cells and to influence the outcome of *H. pylori* infection [12]. IRAK-M^−/−^ DCs however were comparable to WT DCs in generating T_H_17 and T_reg_ in vitro. Additionally, the IRAK-M deficiency in mice did not affect iT_reg_ generation in vivo.

IRAK-M appears to play different roles in other infections and can be either beneficial or deleterious to the host [35], [36], [40], [54], [56]. For example, IRAK-M deficiency led to improved bacterial clearance and host survival in response to both *Streptococcus pneumoniae* and *Klebsiella pneumoniae* infections [35], [36]. In contrast, IRAK-M deficiency is deleterious to the host after influenza infection because the pro-inflammatory phenotype leads to more extensive lung injury in the host [40]. These studies all indicate that IRAK-M helps limit inflammation against pathogenic microbes, an event that can be beneficial to the host, as in the case of influenza infection. Interestingly, gut commensals have also been shown to activate IRAK-M expression and in the absence of both IRAK-M and IL-10, mice were more prone to developing colitis [34].

Of possible relation to *H. pylori* infection, in human genetic studies, IRAK-M has also been associated with asthma in an Italian cohort [57]. The association was not observed in either Japanese or German groups [58], [59]. Given the link between *H.* pylori infection and the reduced incidence of asthma in a variety of studies [24], [27], [32], it will be interesting to further dissect how IRAK-M affects the host response in *H. pylori* infection, and whether it has consequences at other mucosal sites such as the lung. We are currently working on further elucidating the role of IRAK-M in *H. pylori* infection and looking at parameters of the immune response outside of DCs activation.

In summary, we present data to demonstrate that *H. pylori* upregulates IRAK-M expression in DCs. We also show that IRAK-M normally functions to downregulate events associated with immune activation such as MHCII expression and MIP-2 production, and promotes regulatory activity such as the production of IL-10 and expression of PD-L1. IRAK-M expression as well as the activities associated with IRAK-M were dependent upon TLR2, and to a lesser extent TLR4 activation. However, we were unable to demonstrate that IRAK-M plays a role in skewing the balance between T_H_17 and T_reg_ cells. Thus, the manifestation of IRAK-M expression may be in limitations in acute or innate host responses. It will be noteworthy to explore how IRAK-M may affect the variety of disease outcomes in *H. pylori* infection and whether there may be any therapeutic potential in modulating IRAK-M expression.

## Supporting Information

Figure S1 **GM-CSF BMDCs and Flt3L BMDCs share similar cytokine profiles when IRAK-M is deficient.** Supernatant from WT and IRAK-M^−/−^ BMDCs generated by the two different methods stimulated with either live *H. pylori* SS1 (MOI 10) or SS1 and 26695 antigen lysate were collected at 24 h and used to determine TNFα and IL-10 levels by ELISA. Data reflects two independent experiments. Error bars indicate standard deviations. *, *P*<0.05.
(TIF)

Figure S2 **WT and IRAK-M deficient BMDCs have similar T cell differentiation capabilities in the presence of** ***H. pylori*** **stimulation.** BMDCs isolated from WT and IRAK-M^−/−^ mice were plated and pulsed with either media or *H. pylori* SS1 lysate for 2 hours before CD4+ T cells isolated from SS1 infected C56BL/6 animals were added to the wells for 72 hours. Cells were restimulated with PMA and ionomycin in the presence of monesin, and production of (A) IFNγ, (B) IL-17A or (C) Foxp3 in CD4^+^ T cells was measured by flow cytometry.
(TIF)
