# Supplementary figures and images for: IRAK-M Expression Limits Dendritic Cell Activation and Proinflammatory Cytokine Production in Response to Helicobacter pylori
Source: PLoS One. 2013 Jun 11;8(6):e66914. doi: 10.1371/journal.pone.0066914 (PMC3679069; doi:10.1371/journal.pone.0066914)

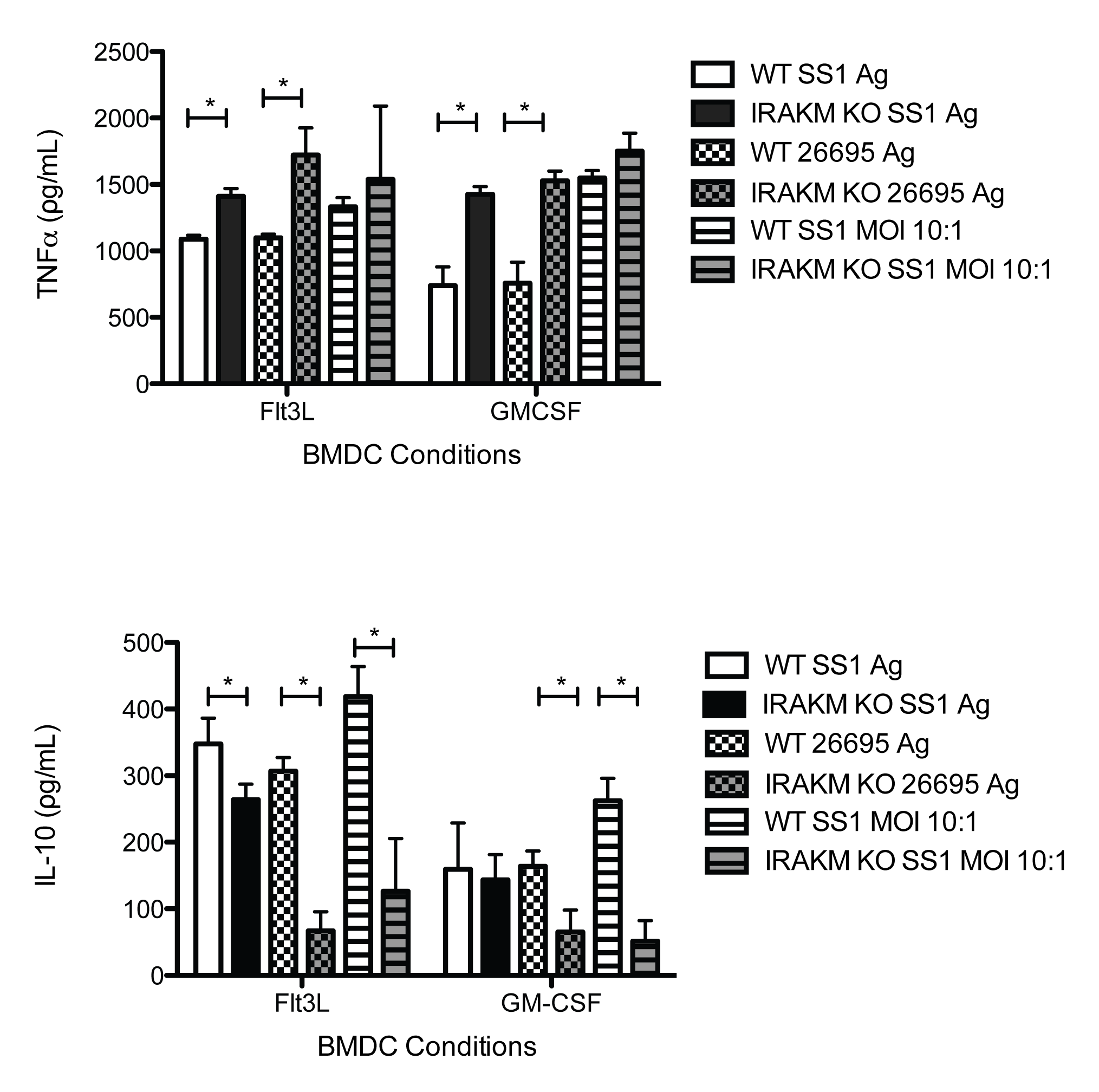

Supplement: Figure S1 — GM-CSF BMDCs and Flt3L BMDCs share similar cytokine profiles when IRAK-M is deficient. Supernatant from WT and IRAK-M−/− BMDCs generated by the two different methods stimulated with either live H. pylori SS1 (MOI 10) or SS1 and 26695 antigen lysate were collected at 24 h and used to determine TNFα and IL-10 levels by ELISA. Data reflects two independent experiments. Error bars indicate standard deviations. *, P<0.05. (TIF) [file pone.0066914.s001.tif]

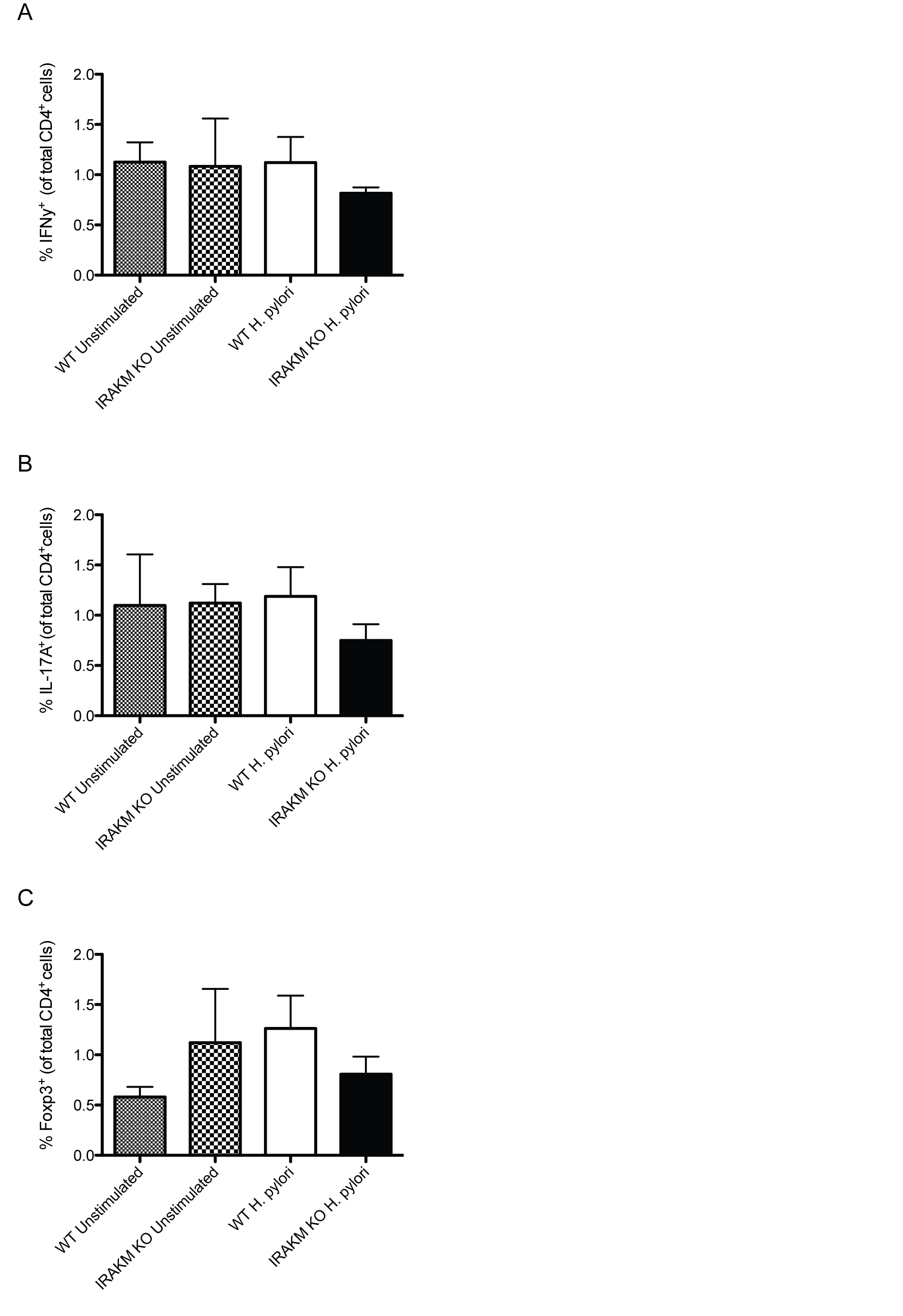

Supplement: Figure S2 — WT and IRAK-M deficient BMDCs have similar T cell differentiation capabilities in the presence of H. pylori stimulation. BMDCs isolated from WT and IRAK-M−/− mice were plated and pulsed with either media or H. pylori SS1 lysate for 2 hours before CD4+ T cells isolated from SS1 infected C56BL/6 animals were added to the wells for 72 hours. Cells were restimulated with PMA and ionomycin in the presence of monesin, and production of (A) IFNγ, (B) IL-17A or (C) Foxp3 in CD4+ T cells was measured by flow cytometry. (TIF) [file pone.0066914.s002.tif]
